# Effect of Ultrasonic Condensation Time on Void Formation and Microhardness of Well-Root^TM^ PT Apical Plugs in 3D-Printed Immature Teeth

**DOI:** 10.3390/ma18214835

**Published:** 2025-10-22

**Authors:** Krasimir Hristov, Ralitsa Bogovska-Gigova

**Affiliations:** Department of Pediatric Dentistry, Faculty of Dental Medicine, Medical University of Sofia, 1431 Sofia, Bulgaria; r.bogovska@fdm.mu-sofia.bg

**Keywords:** apical plug, Well-Root^TM^ PT, ultrasonic activation, micro-CT, microhardness, immature teeth

## Abstract

Background: This study aimed to evaluate the impact of varying durations of ultrasonic condensation on the formation of internal and external voids and the microhardness of apical plugs created with premixed bioceramic putty Well-Root^TM^ PT in standardized 3D-printed immature permanent teeth using micro-CT imaging and Vickers microhardness testing. Methods: Forty-eight 3D-printed upper incisors with simulated open apices (2 mm canal diameter) were divided into four groups (*n* = 12 each) based on apical plug condensation technique as follows: Group 1 (control, manual condensation), Group 2 (3-s Ultrasonic at 25 kHz), Group 3 (9-s Ultrasonic at 25 kHz), and Group 4 (15-s Ultrasonic at 25 kHz). Well-Root^TM^ PT was used to form 5 mm apical plugs under a microscope. Samples were stored at 37 °C and 100% humidity for one week. Micro-CT imaging was used to quantify internal, external, and total void volumes (% of total material volume), while microhardness was measured using a Vickers tester (1 kgf load, 10 s) on polished apical plug sections. Statistical analysis was performed using ANOVA and Tukey post hoc tests. Results: Group 4 (15-s Ultrasonic) exhibited significantly higher external and total void volumes compared to Groups 1–3 (*p* < 0.001), with no significant differences in internal voids across groups (*p* > 0.05). Microhardness was highest in Group 1 (mean VHN: 76.95 ± 3.73), followed by Group 2 (73.11 ± 4.82), Group 3 (55.11 ± 5.28), and Group 4 (51.25 ± 7.73) (*p* < 0.05). Shorter ultrasonic durations (3-s Ultrasonic) resulted in fewer voids and higher microhardness compared to longer durations (15-s Ultrasonic). There was no statistically significant difference in void size among the groups compared (*p* > 0.05). Fractal dimension analysis showed that prolonged ultrasonic condensation results in less complex voids compared to shorter activation. Conclusion: Manual condensation of premixed bioceramic putty, by promoting denser particle packing without ultrasonic-induced disruptions, leads to higher microhardness. Brief ultrasonic activation (3-s Ultrasonic) optimizes the quality of Well-Root^TM^ PT apical plugs by minimizing voids and maintaining higher microhardness, thus enhancing the apical seal. Prolonged ultrasonic activation (15-s Ultrasonic) increases void formation and reduces microhardness, potentially compromising the long-term integrity of the apical barrier.

## 1. Introduction

Permanent teeth with immature roots are characterized by a wide apical foramen due to incomplete root development and apical closure [1]. The root canal walls in these teeth are thin and fragile, particularly in the cervical and middle thirds, making them more susceptible to fracture [2]. The root length is shorter than that of fully developed teeth, and the canal is wider and more divergent, with a flared apex [3]. The dentinal walls are not fully thickened, and the pulp chamber is relatively large due to ongoing dentinogenesis [1]. Clinical management must account for their structural vulnerability and the potential for continued development if the pulp can be preserved or regenerated [2]. These features significantly influence the management of pulp and periapical pathology [4,5].

Immature roots are found in recently erupted teeth still undergoing root maturation, a process that can be disrupted by traumatic injury or caries [6]. If the pulp remains vital, continued root development (apexogenesis) can lead to apical closure and thickening of the root walls [5]. If the pulp becomes necrotic, root development ceases, requiring apexification or regenerative endodontic procedures to promote further maturation and strengthen the root structure [6]. The primary goal of apexification is to create an artificial apical barrier to enable effective obturation and prevent extrusion of filling materials, particularly when regenerative procedures are not feasible or when significant coronal structure loss necessitates a post and core restoration [4,7]. Mineral trioxide aggregate (MTA) is the gold standard for apexification due to its high success rates and biocompatibility [8,9,10].

The clinical protocol for apexification includes thorough canal disinfection, typically with sodium hypochlorite irrigation and, if indicated, interim calcium hydroxide dressing. A 3–5 mm apical plug is placed orthogradely, incrementally packed, and condensed, with a moist cotton pellet placed to allow for setting. The remainder of the canal is filled with gutta-percha or another suitable material, and the tooth is restored [4,11]. Ultrasonic activation during apical plug placement improves material adaptation to dentin walls, reduces porosity, and forms a denser, more uniform apical barrier, minimizing microleakage risk [12,13,14]. Studies show that ultrasonic activation (10–20 s via an endodontic tip) enhances bond strength and marginal adaptation, particularly for Biodentine™ (Septodont, Saint-Maur-des-Fossés, France), and reduces porosity in MTA and BioAggregate (Innovative BioCeramix, Vancouver, Canada) [12,13,14,15]. Biodentine™ shows good adaptation regardless of technique, while MTA benefits most from mechanical mixing and ultrasonic activation to minimize voids [12,13,14]. However, prolonged ultrasonic activation (>10 s) may increase porosity and reduce microhardness in MTA [16].

Well-Root™ PT (Vericom, Chuncheon-si, Republic of Korea) is a premixed, injectable calcium silicate-based bioceramic material used for apical plug formation in immature permanent teeth. It is insoluble, dimensionally stable, and highly alkaline [17]. Well-Root™ PT exhibits short-term sealing ability comparable to MTA, with reduced nanoleakage in the first week and similar dentin wall adaptation when used as a retrograde filling material [18]. The material promotes cell adhesion and favorable cell morphology, indicating excellent biocompatibility [19]. It also demonstrates high radiopacity and microhardness comparable to or surpassing MTA, supporting its use in apexification [20]. Well-Root™ PT shows clinical and radiographic success rates similar to MTA, with advantages like ease of use and no tooth discoloration [20,21]. Limited data exist on void formation with Well-Root™ PT compared to other calcium silicate-based materials using various application techniques. As a premixed putty, Well-Root™ PT exhibits reduced technique sensitivity and, with advanced placement methods, is faster and less prone to nanoleakage than MTA [13]. The impact of ultrasonic activation on Well-Root™ PT apical plug quality remains understudied.

Ultrasonic activation of bioceramic materials like Well-Root™ PT involves complex mechanical interactions that can influence microstructure and lead to volumetric or surface voids [22]. Ultrasonic energy generates acoustic microstreaming and cavitation, promoting particle dispersion and material flow but potentially disrupting particle packing [23], particularly in premixed putties containing calcium aluminosilicate, zirconium oxide, calcium silicate, calcium sulfate, and thickening agents [13,16]. These components’ distinct physical properties (e.g., particle size, density, viscosity) may respond variably to ultrasonic energy, potentially causing microcavitation or shear stresses that create voids, which can compromise the apical seal and increase microleakage risk [24]. Calcium silicate-based cements hydrate to form calcium silicate hydrate (C-S-H) and calcium hydroxide, with voids arising from incomplete hydration, water evaporation, or air entrapment [25]. Microstructure and porosity depend on particle size, water-to-powder ratio, and additives [26,27]. Optimized particle size and additives (e.g., dispersants, plasticizers) reduce voids and enhance mechanical strength, while higher water content or poor mixing increases porosity [26,28].

Zirconium oxide, used as a radiopacifier and inert filler, does not participate in hydration, but affects packing density and cement flow. Properly dispersed, it reduces agglomeration and voids, but poor mixing can increase porosity [29,30]. Calcium sulfate, a void filler and grafting material, sets by crystallization, with voids forming if condensation is inadequate or liquid content is excessive [31]. Its resorption and mechanical stability are influenced by initial porosity, minimized by careful handling [32]. Thickeners’ rheological properties affect mixture viscosity and stability, reducing void risk with uniform mixing. Gum-based thickeners provide more stable viscosity than starch-based ones, which may increase porosity if mishandled [33]. Optimizing particle size, water content, mixing techniques, and additives minimizes void formation, impacting clinical performance.

Microhardness, measured using the Vickers test, is critical for apical plug materials, as it correlates with resistance to deformation, durability, and sealing ability, essential for long-term endodontic success [34]. Biodentine™ exhibits the highest microhardness, followed by Well-Root™ PT, with MTA showing the lowest [34]. Higher microhardness enhances fracture resistance, marginal adaptation, and resistance to functional stresses [35]. Brief ultrasonic activation (2–8 s) optimizes MTA’s microhardness by increasing density and reducing voids, but prolonged ultrasonication (>10 s) increases porosity and reduces microhardness [16,36]. Biodentine™ and Well-Root™ PT maintain high microhardness, regardless of placement technique [35]. 

The purpose of this study was to assess how varying durations of ultrasound condensation affect the formation of internal and external voids, as evaluated by micro-CT imaging, as well as the microhardness of apical plugs from premixed bioceramic putty Well-Root PT™. The insights gained from this study aim to optimize the quality and integrity of the apical seal. The null hypothesis was that the duration of ultrasound condensation would have no impact on the quality and microhardness of the created apical plugs.

## 2. Materials and Methods

### 2.1. Sample Size Calculation

Based on previous studies from the specialized literature [17,37], and using G-Power statistical power analysis software (version 3.1.9.7), a total sample size of *n* = 48, divided into 12 per group, was calculated to be adequate to detect a large effect size (W = 0.48) with a statistical power (1-β error) of 0.95 (95%) and a significance level (α error) of 0.05 (5%) for a hypothesis test.

### 2.2. Sample Selection

Standardized 3D-printed upper incisors with endodontic access cavities and 21 mm in length (DRSK, Hässleholm, Sweden) were used in the study. These models were designed to simulate incomplete root development; they exhibited divergent apical root walls and featured a canal diameter of 2 mm at the apex. All procedures were performed by a single experienced endodontist. To ensure calibration, the clinician underwent a standardized protocol prior to the study, which included a series of mock procedures using the same 3D-printed teeth to refine techniques specific to the study’s methodology. This calibration process involved performing 10 simulated apexification procedures under controlled conditions, with outcomes evaluated by two independent senior endodontists for consistency in material placement, sealing quality, and adherence to the study protocol. The teeth were fixed in an endodontic system for training (Protrain, Simit Dental Srl, Mantova, Italy) with silicone impression material Apical plugs were created under microscope (Zumax OMS1800, Zumax Medical, Jiangsu, China) by a single clinician with Well-Root™ PT (Vericom, Chuncheon, Republic of Korea). The material was compacted using appropriately sized pluggers (Dentsply Sirona, Bensheim, Germany) to a thickness of 5 mm in the apical region. The distribution of the samples based on the apical plugs condensation methods is presented in Table 1. A standardized ultrasonic tip (size 20, non-cutting, stainless steel) and a fixed 25 kHz power setting were used for all ultrasonic activation groups, with procedures performed by a single operator to minimize variability. Any excess material in the apical area was removed with a sterile blade if overfilling occurred. All samples were stored in an incubator at 37 °C and 100% relative humidity for one week to ensure complete setting and to replicate internal oral conditions.

### 2.3. Scanning Procedure

The samples were scanned to measure the percentage of internal, external, and total voids within the apical plug using a high-resolution desktop micro-CT (Computerized Tomography) scanner (SkyScan 1272, Bruker, Kontich, Belgium). Plastic immature teeth from all groups were securely placed in a mold to ensure consistent alignment during the scanning process. The scanning parameters included a voltage of 85 kV, a current of 117 μA, a 1.0 mm aluminum filter, a pixel size of 9 μm, a rotation step of 0.9°, a complete 360° sample rotation, frame averaging of 4, and an exposure time of 140 ms for each projection. Each sample scan took approximately 10 min. The NRecon software (version 2.2.0.6, Bruker, Kontich, Belgium) was used to reconstruct tooth projections into cross-sectional slices, with the following settings: ring artifacts and smoothing set to 0, beam hardening at 55%, and misalignment compensation applied.

### 2.4. Evaluation of Internal and External Voids Using Micro-CT

The reconstructed specimens were analyzed using CTAn visualization software (version 1.23.01, Bruker, Kontich, Belgium) to assess the 3D microarchitecture of the apical plugs. Global thresholding was applied by visual matching with greyscale images. The same global threshold values were applied to all samples. Distinct segmentation was used to differentiate voids from the materials of the apical plugs, based on a micro-CT resolution of 9 μm. Voids were defined as air pockets or gaps within or at the surface of the apical plug, with a minimum detectable size of approximately 9 μm due to the scanner’s pixel size. The fractal dimension was determined in three dimensions using the Kolmogorov box-counting method [38].

Internal voids were defined as air pockets fully enclosed within the Well-Root™ PT material, with no connectivity to the external surface, as confirmed by 3D reconstructions using CTAn software (version 1.23.01, Bruker, Kontich, Belgium). External voids were defined as gaps at the interface between the apical plug and the 3D-printed root canal walls, exhibiting direct connectivity to the external surface. To address potential ambiguity regarding deep surface holes, we further classified voids as external if they originated at the surface and extended into the plug body, regardless of depth, provided they maintained connectivity to the external environment. Internal voids with connectivity to the surface (e.g., through microchannels or fissures) were reclassified as external voids during 3D analysis to ensure accurate differentiation. The micro-CT resolution of 9 μm limited the detection of smaller microchannels, but no such connectivity was observed in our samples based on visual inspection of 3D reconstructions. Void dimensions (volume) were quantified where possible, with representative void sizes visualized. After thresholding and binarization, quantitative analysis was performed to calculate internal, external, and total void volumes as percentages of the total material volume. A single examiner, calibrated through standardized training on CTAn software (version 1.23.01) and Vickers microhardness testing protocols, performed all micro-CT and microhardness evaluations to ensure consistency in quantitative measurements.

### 2.5. Microhardness Testing

Teeth samples were mounted in custom-designed holders to ensure stability during testing. Each tooth was vertically positioned and embedded in epoxy resin in the coronal part to maintain consistent orientation. To prepare a flat and uniform testing surface, a 1 mm section from the apex of each tooth was cut. This step ensured that the surface was level and parallel to the testing plane, minimizing variations in measurements due to surface irregularities. The surfaces of these slices were rinsed with normal saline for 1 min to remove any remaining debris. This was followed by polishing the surfaces using 300 to 1200 grit paper.

**Figure 1:**
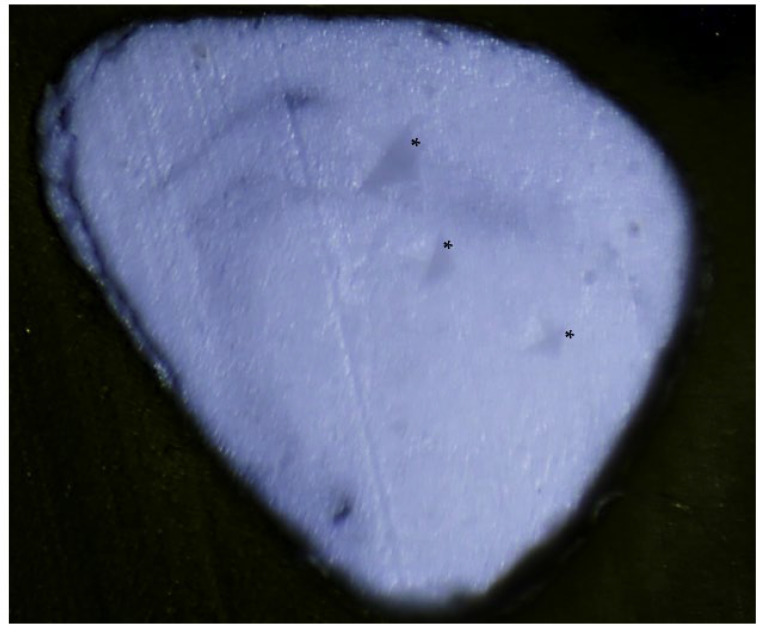
Cut sample under microscope with marked indentations (asterisks).

Surface microhardness was measured with an automatic Vickers microhardness tester (Indentec hardness testing, Zwick Roell, Ulm, Germany). A diamond indenter with a square-based pyramidal shape was used to apply a load to the surface of the apical plug. Three indentations were made on the surface of each sample, using a 1 kgf load applied for 10 s, with the indenter tip positioned parallel to the surface. The indentations were taken for each sample at intervals of 500, 1000, and 1500 μm. The lengths of the indentations were measured microscopically (Figure 1).

The Vickers Hardness Number (VHN) was calculated using the formula VHN = 1.854/d_1_ × d_2_, where 1.854 represents the applied force in kg/m^2^ and d_1_ and d_2_ are the mean diagonal length of the indentation.

A 1 kgf load was selected for Vickers microhardness testing based on established protocols for calcium silicate-based cements, which typically exhibit sufficient mechanical strength to withstand this load without surface damage [34].

### 2.6. Statistical Analysis

The data followed a normal distribution (Shapiro–Wilk test, *p* > 0.05) with homogeneous variances (Levene’s test, *p* > 0.05). A one-way ANOVA was conducted to assess the percentages of internal, external, and total voids and microhardness. Tukey’s test was applied for multiple comparisons within the different groups. To assess the relationship between the duration of ultrasonic condensation and microhardness of the bioceramic putty, the Pearson correlation coefficient was calculated. Statistical analyses were performed using SPSS, Version 19.0 (IBM Corp., Armonk, NY, USA). Differences among experimental groups were considered significant at *p* < 0.05.

## 3. Results

Representative microscopic images of the indented surfaces (Figure 1) confirmed no visible cracking or deformation across groups, supporting the integrity of the measurements.

**Figure 2:**
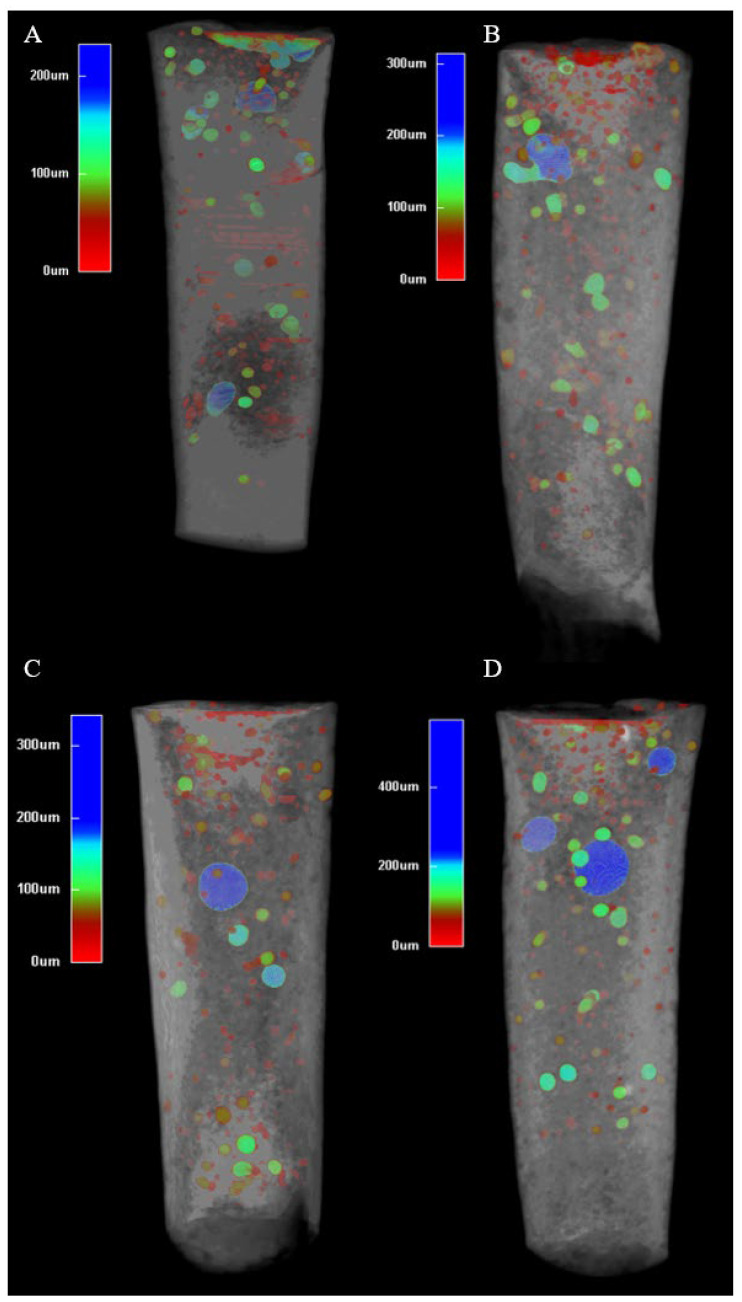
Representative micro-CT images of apical plugs, created with different condensation techniques with superimposed voids (color-coded depending on their size). (**A**) Manual technique; (**B**) ultrasound condensation for 3 s; (**C**) ultrasound condensation for 9 s; (**D**) ultrasound condensation for 15 s.

Figure 2 shows representative micro-CT images of the apical plugs for each group, illustrating the distribution of the voids.

**Figure 3:**
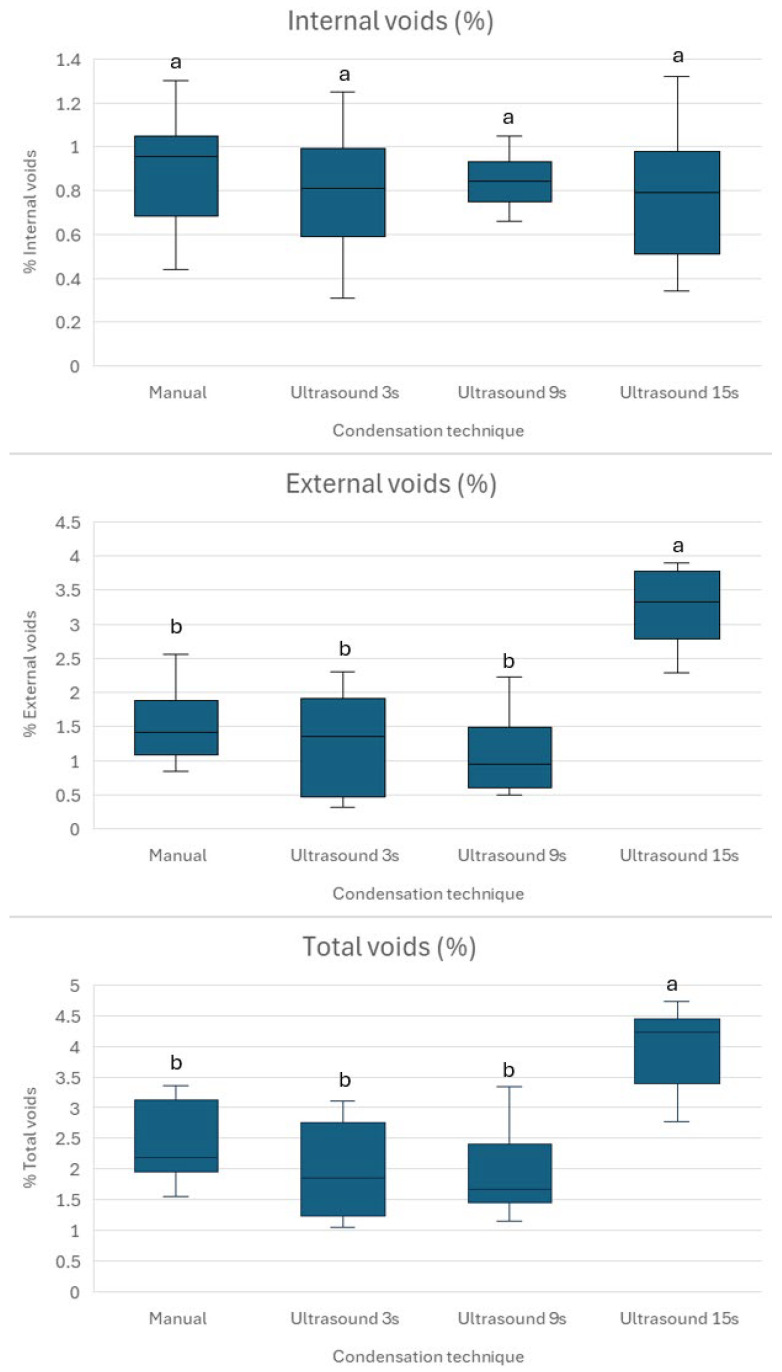
Internal, external, and total voids as a percentage of total material volume. Different lowercase letters (a,b) indicate significant differences (*p* < 0.05).

In Groups 1–3, the plugs exhibited a relatively uniform structure with small, scattered internal voids and minimal external voids along the root interface. In contrast, Group 4 (15-s Ultrasonic) exhibited larger voids evident in the material. These visual differences align with the quantitative data in Figure 3, where Group 4 (15-s Ultrasonic) showed significantly higher external and total void volumes (*p* < 0.001).

The results from Figure 3 indicate varying levels of internal, external, and total voids across the four groups, with Group 4 showing notably higher external and total voids (3.23 ± 0.59% and 4.04 ± 0.70%, respectively) compared to Groups 1–3, which share similar ranges for internal (0.81–0.91%) and external voids (1.27–1.54%). The statistic tests reveal no significant differences in internal voids between groups (*p* > 0.05), suggesting comparable internal porosity. However, for external and total voids, Group 4 significantly differs from all other groups (*p* < 0.001), indicating a distinct characteristic due to the duration of the ultrasonication (15 s). The mean void size in the apical plug was 115.28 ± 38.64 µm after manual condensation, 109.43 ± 23.8 µm following 3 s ultrasonic activation, 146.49 ± 36.48 µm after 9 s activation, and 176.85 ± 61.28 µm after 15 s activation. There was no statistically significant difference in void size among the groups compared (*p* > 0.05).

**Figure 4:**
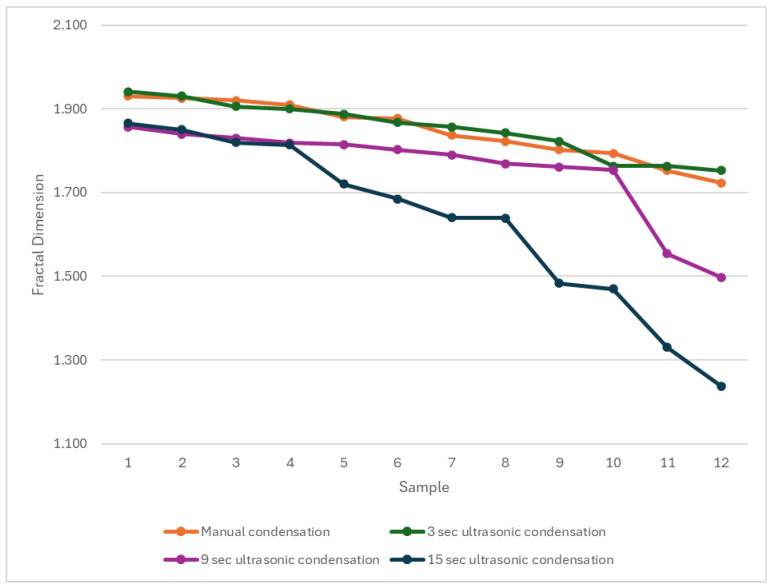
Fractal dimension analysis of formed voids after manual or ultrasonic condensation.

The analysis of void fractal dimensions (Figure 4) showed mean fractal dimension of 1.84 ± 0.07 for manual condensation, 1.85 ± 0.06 for ultrasonic activation at 3 s, 1.75 ± 0.11 for ultrasonic activation at 9 s, and 1.62 ± 0.12 for ultrasonic activation at 15 s. Statistical analysis indicated a significant difference when comparing manual and 3 s ultrasonic condensation with 15 s condensation (*p* = 0.001). No statistically significant differences were observed when comparing other groups (*p* > 0.05).

**Figure 5:**
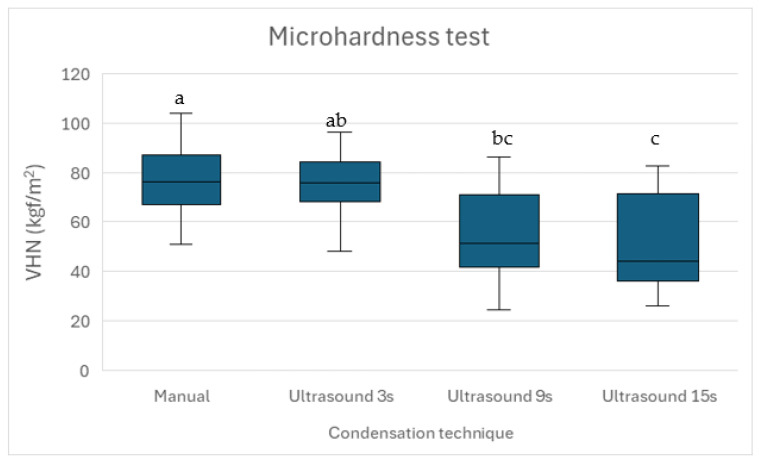
Microhardness values (VHN, Vickers Hardness Number) of the studied groups. Different lowercase letters (a–c) indicate significant differences (*p* < 0.05).

The microhardness test indicated that ultrasonic condensation reduced the material’s mechanical properties, with longer ultrasonication resulting in lower microhardness values (Figure 5). Significant differences were observed between manual condensation and ultrasonic activation for 9 s and 15 s (*p* = 0.039 and *p* = 0.011, respectively), indicating that microhardness decreases with the increase in the duration of the ultrasonic activation. No significant differences were detected between manual and ultrasonic condensation for 3 s (*p* = 0.962), indicating that manual condensation and brief ultrasonication provide similar microhardness values of bioceramic putties.

Pearson correlation indicated no link between total voids and microhardness after manual condensation (r(10) = 0.124, *p* = 0.70), or ultrasonic condensation for 3 s (r(10) = 0.115, *p* = 0.72) and 9 s (r(10) = 0.446, *p* = 0.07). Significant correlation was found with 15 s of ultrasonic condensation (r(10) = 0.747, *p* = 0.02).

## 4. Discussion

This study investigates the impact of varying durations of indirect ultrasonic activation on the quality of apical plugs formed with Well-Root™ PT, focusing on void formation and microhardness. The findings indicate that varying the duration of ultrasonic activation affects the quality of apical plugs made with premixed bioceramic putty. Specifically, it increases the percentage of external voids and decreases the microhardness while having no significant impact on the percentage of internal voids. Thus, the null hypothesis was partially rejected. The management of necrotic immature permanent teeth with open apices poses a significant challenge in endodontics due to the absence of an apical constriction, which complicates effective obturation and apical sealing [4,5,7]. The results offer valuable insights into optimizing apical plug placement techniques to enhance the long-term integrity of the apical seal.

The selection of the three time intervals for ultrasonic activation—3, 9, and 15 s—was strategically designed to explore a range of durations, encompassing both shorter and intermediate times relative to the commonly reported 10–20 s range for ultrasonic activation of calcium silicate-based materials [12,14]. The 3 s interval was chosen to evaluate the efficacy of brief ultrasonic activation, which may minimize potential adverse effects, such as excessive heat generation or material disruption, while still promoting material flow and compaction. This short duration aligns with prior studies suggesting that brief ultrasonication (2–8 s) optimizes the density and reduces voids in apical plugs [16]. The 9 s interval was selected as an intermediate duration to assess whether a moderate increase in activation time could further enhance material adaptation or inadvertently introduce negative effects, such as increased porosity, as observed with prolonged ultrasonication. Finally, the 15 s interval was included as it falls within the higher end of the conventional 10–20 s range, serving as a reference to compare the effects of longer durations against a standard protocol. This systematic selection allowed us to investigate the dose–response relationship between ultrasonic activation duration and the quality of Well-Root™ PT apical plugs, specifically in terms of void formation and microhardness. Our findings, which indicate that 3 s of ultrasonic activation balances low void volumes with high microhardness, provide evidence to support the use of brief activation durations in clinical practice to optimize the apical seal in immature permanent teeth.

Density and porosity are critical parameters that affect the success of endodontic treatments [17]. Porosity is an intrinsic characteristic of tricalcium silicate cement, arising from gaps between non-hydrated cement particles. During the hydration process, these gaps initially become filled with water. As hydration progresses, the reaction products gradually fill these voids, reducing overall porosity. This reduction is vital for enhancing the material’s sealing capability and, consequently, the effectiveness of the treatment [17].

Voids play a crucial role in the effectiveness of apical plugs in immature permanent teeth because they can compromise the seal and integrity of the apical barrier. This increases the risk of microleakage, bacterial invasion, and potential endodontic treatment failure. When voids are present within or near the apical plug, they can allow periapical fluids and microorganisms to enter the canal system, leading to persistent periapical inflammation or reinfection. This jeopardizes the healing process in the periapical area and the long-term retention of the tooth [24].

Ultrasonic activation modifies the microstructure of premixed bioceramics in dental and endodontic applications by enhancing particle dispersion, reducing porosity, and improving adaptation to dentin walls [13]. The mechanism involves ultrasonic energy generating acoustic microstreaming and localized shear forces, which disrupt particle agglomerates, resulting in a more homogeneous and compact material with fewer internal voids and better marginal adaptation to canal walls [12,39]. Micro-CT and SEM analyses show that ultrasonic activation promotes uniform particle distribution and denser packing in tricalcium silicate-based cements, such as MTA and BioAggregate, compared to manual or bulk placement techniques. This reduces porosity and enhances condensation quality, critical for the sealing ability and long-term stability of root-end fillings. However, excessive ultrasonication may increase porosity and reduce microhardness, highlighting the need for optimal activation times to balance improved adaptation with material integrity [16]. While ultrasonic activation does not significantly alter the chemical composition of bioceramics, it can affect surface characteristics, particularly if applied before adequate setting. The primary mechanisms—acoustic microstreaming and shear-induced particle redistribution—yield a denser, less porous, and better-adapted bioceramic microstructure [40]. These mechanisms explain why brief ultrasonic activation enhances Well-Root™ PT’s density and sealing ability, while prolonged activation increases voids.

The durations of ultrasonic activation—3, 9, and 15 s—were selected to systematically evaluate the dose–response relationship between activation time and apical plug quality, based on prior studies of calcium silicate-based materials [12,13,14,16]. The 3 s duration was chosen to represent brief activation within the optimal range (2–8 s) reported to minimize void formation and enhance material density [16]. The 9 s duration was selected as an intermediate time to assess whether moderate increases in activation could further improve material adaptation or introduce adverse effects, such as increased porosity. The 15 s duration was included as it falls within the conventional 10–20 s range used in clinical protocols for calcium silicate-based materials, serving as a reference for prolonged activation [12,13,14]. These durations were chosen to balance clinical applicability with the need to investigate the effects of ultrasonic energy on Well-Root^TM^ PT’s microstructure, but further studies with additional intermediate durations (e.g., 5, 7, or 12 s) are warranted to refine the optimal activation time.

Prolonged ultrasonic activation (15 s) likely increases external voids due to excessive acoustic microstreaming and cavitation, which disrupt the packing of Well-Root™ PT’s components, such as calcium aluminosilicate and zirconium oxide, leading to gaps at the material–dentin interface [16,23]. In contrast, brief 3 s activation enhances particle dispersion and material flow, promoting better adaptation to dentin walls and minimizing void formation [12,13]. The consistent internal microstructure across all groups (Figure 3) suggests that Well-Root™ PT’s premixed formulation ensures uniform hydration, likely due to its optimized viscosity and reduced technique sensitivity compared to manually mixed materials like MTA [13,15]. Clinically, increased external voids with prolonged activation may weaken the apical seal, elevating the risk of microleakage and bacterial infiltration, which could lead to periapical inflammation or treatment failure [24]. Conversely, the lower void volumes and higher microhardness achieved with 3 s activation (Figure 3 and Figure 4) support a denser, more durable apical barrier, enhancing the long-term prognosis of endodontically treated immature permanent teeth. These findings underscore the importance of optimizing ultrasonic activation duration to balance material compaction with marginal adaptation, ensuring effective sealing and mechanical stability.

To achieve an optimal apical plug placement, it is essential to have a dense, void-free fill, ensuring a reliable apical seal. Research indicates that delivery techniques that minimize voids, such as modified cannula, result in significantly less microleakage compared to traditional methods [24]. This highlights the clinical importance of avoiding voids during the formation of apical plugs, especially in immature teeth [24]. In such cases, the absence of an apical constriction makes achieving a hermetic seal more challenging [41]. This study shows that no placement technique is currently able to create void-free apical plugs (Figure 2). Brief ultrasonic activation for 3 s minimizes void formation and maintains higher microhardness compared to longer durations of 9 and 15 s. Specifically, 15 s ultrasound condensation resulted in significantly higher external and total void volumes (*p* < 0.001), suggesting that prolonged ultrasonic activation may disrupt the material’s homogeneity and its adaptation to dentin walls. This aligns with previous research, which reported that excessive ultrasonication (beyond 10 s) increases porosity and void formation in calcium silicate-based materials, such as mineral trioxide aggregate (MTA), due to over-vibration and potential microcavitation effects [16,36]. In contrast, shorter ultrasonic durations (between 2 and 8 s) improve material compaction and flow, resulting in denser plugs with fewer voids [16,36].

The absence of statistically significant differences in internal void volumes across groups (*p* > 0.05) suggests that Well-Root™ PT, as a premixed bioceramic putty, produces comparable internal void volumes regardless of the condensation technique. This may be attributed to its injectable consistency and reduced technique sensitivity compared to MTA, which requires manual mixing and is more prone to voids when hand-condensed [13,15]. However, the increased external voids in Group 4 suggest that prolonged ultrasonic activation may compromise the material’s uniformity and dentin wall adaptation, potentially elevating the risk of microleakage and treatment failure. This finding emphasizes the need to balance material compaction with marginal adaptation by optimizing ultrasonic activation parameters. The results are consistent with another study, which reported that indirect ultrasonic activation of premixed calcium silicate cement led to increased quantitative leakage [42]. The same study did not find any significant difference in void formation after ultrasonic activation. This may be attributed to the use of two-dimensional periapical radiograph, not used in the current study, which used more precise micro-CT assessment.

While the percentage void volumes reported in this study (1–5%) are relatively low, the size and distribution of voids are likely more critical to the mechanical strength of Well-Root™ PT apical plugs, as they can act as stress concentration points that compromise microhardness and fracture resistance [43,44]. Larger voids, especially those at the interface, can facilitate crack propagation under functional loads, reducing the long-term integrity of the apical barrier. In contrast, smaller and more evenly distributed voids may minimize stress concentration. These findings suggest that optimizing ultrasonic activation to reduce voids and achieve a uniform distribution, as seen with brief activation (3 s), is crucial for enhancing the mechanical strength of apical plugs. However, the current study’s reliance on percentage void volume limits the ability to fully characterize the mechanical implications of void morphology (including their size, shape, and location). Future studies should employ mechanical testing (compressive strength and fracture toughness) alongside advanced imaging to correlate void size, distribution, and mechanical performance, ensuring a comprehensive understanding of their impact on clinical outcomes.

The fractal dimension analysis provides additional insight into the microstructural effects of ultrasonic condensation on Well-Root™ PT apical plugs by quantitatively characterizing the homogeneity and complexity of pore or void distributions within the material. The use of fractal dimension and multifractal spectral symmetry allows for a more nuanced assessment of pore structure than conventional metrics, as demonstrated in the ultrasonic characterization of heterogeneous media, where lower fractal dimension values are associated with less complex, more uniform void structures [45]. The results in Figure 5 suggests that prolonged ultrasonic condensation results in less complex voids compared to shorter activation. The significantly lower fractal dimension in Group 4 (15 s ultrasonic activation) suggests less complex void structures, potentially indicating larger, more uniform voids that could act as stress concentration points, compromising mechanical stability. This aligns with the increased external void volumes and reduced microhardness observed in Group 4, reinforcing the conclusion that prolonged ultrasonic activation is detrimental to apical plug quality. In contrast, the similar fractal dimensions in Groups 1 and 2 (manual and 3 s ultrasonic activation) indicate comparable void complexity, supporting the use of brief ultrasonic activation to achieve a dense, well-adapted apical barrier. These findings highlight the importance of optimizing condensation techniques to balance void morphology and material integrity, enhancing the long-term prognosis of endodontically treated immature teeth. The relationship between fractal parameters and ultrasonic attenuation further substantiates the utility of fractal analysis in evaluating the microstructural integrity of apical plugs subjected to ultrasonic condensation.

This is the first study to evaluate the effect of ultrasonic condensation during apical plug formation on the microhardness of premixed bioceramic putty Well-Root™ PT. Microhardness is a crucial indicator of a material’s durability and resistance to deformation. The present study found that manual condensation had the highest microhardness values, consistent with previously reported data [46]. The microhardness values decreased progressively with longer ultrasonic durations, with Group 4 exhibiting the lowest values (*p* < 0.05). Microscopic examination of the indented surfaces (Figure 1) showed no visible cracking or deformation, supporting the suitability of this load for Well-Root™ PT. However, lower loads (100–500 g) are recommended for some brittle biomaterials to minimize the risk of microcracking [35], and future studies could validate our findings using a range of loads to ensure measurement reliability. To assess the material’s response to loading and unloading, indentation surfaces were examined microscopically post-testing to evaluate surface integrity and deformation characteristics.

The loading and unloading behavior of Well-Root™ PT, as inferred from indentation surface analysis, indicates that brief ultrasonic activation (3 s) and manual condensation preserve surface integrity, as evidenced by sharper indentation edges and higher microhardness values. Prolonged ultrasonic activation (15 s) likely increases surface porosity, reducing resistance to deformation and contributing to lower microhardness (*p* < 0.05). These findings underscore the importance of optimizing condensation techniques to maintain surface mechanical properties, which are critical for resisting functional stresses and ensuring a durable apical seal.

Previous studies have reported similar findings for MTA, indicating that brief ultrasonic activation (2–8 s) enhances microhardness by improving material density [16,36]. In contrast, prolonged activation (greater than 10 s) decreases microhardness due to increased porosity [16,36]. The high microhardness observed in Group 1 may reflect the stable, dense structure achieved through controlled manual condensation, even though this technique resulted in slightly higher void volumes compared to Group 2. The performance of Group 2, which utilized 3 s of ultrasonic activation, effectively balanced low void volumes with relatively high microhardness, indicating its potential as an optimal technique for Well-Root™ PT apical plugs.

Our findings on Well-Root™ PT’s void formation and microhardness can be contextualized by comparing them with those for MTA and Biodentine™, two widely used calcium silicate-based materials for apexification. Similarly to our results, studies on MTA show that brief ultrasonic activation (2–8 s) reduces void formation and enhances microhardness by improving material density, whereas prolonged activation (>10 s) increases porosity and reduces microhardness, aligning with the higher external voids and lower microhardness in our 15-s Ultrasonic group [16,36]. However, Well-Root™ PT’s premixed formulation exhibited consistent internal void volumes across all groups (*p* > 0.05), unlike MTA, which is more prone to internal voids when manually mixed and condensed due to technique sensitivity [13,15]. Biodentine™, noted for high microhardness and low technique sensitivity, maintains superior adaptation to dentin walls regardless of condensation method, outperforming Well-Root™ PT in microhardness (mean VHN: ~85–90 vs. 76.95 for Manual Condensation) but showing comparable sealing ability [34]. The 3-s Ultrasonic group’s low void volumes and high microhardness (73.11 ± 4.82 VHN) suggest Well-Root™ PT performs comparably to Biodentine™ under optimized conditions, offering clinical advantages like ease of use and no discoloration [21]. Future studies directly comparing these materials under identical protocols are needed to quantify relative performance in vivo.

The clinical implications of these findings are significant. A dense and well-adapted apical plug with high microhardness is essential for preventing bacterial infiltration and ensuring the long-term success of endodontic treatment in immature teeth [24]. The increased void formation and reduced microhardness associated with prolonged ultrasonic activation (15 s) could compromise the apical seal, potentially leading to periapical inflammation or treatment failure. Conversely, brief ultrasonic activation (3 s) appears to enhance the quality of the apical barrier, both in terms of void formation and mechanical properties, thus supporting its use in clinical practice. These findings also suggest that Well-Root™ PT, known for its favorable handling properties and biocompatibility, is suitable for forming apical plugs, particularly when combined with optimized condensation techniques. Meanwhile, although complete void elimination may not be achievable or desirable, controlling the size and distribution of voids could be crucial for optimizing the balance between sealing efficacy and tissue integration [47].

Abedi-Amin et al. evaluated novel calcium silicate/calcium phosphate cements, including experimental calcium silicate cement and two light-curing cements, which demonstrated suitable in vitro sealing ability, biocompatibility, and mineral precipitation as root-end filling materials [48]. Well-Root™ PT’s low void volumes with 3 s ultrasonic activation align with the aforementioned cements’ effective sealing after 2–4 weeks [31]. Its bioactivity, promoting cell adhesion and mineralization [19,20], matches the mineral precipitation observed across these cements, indicating its potential for apatite formation in clinical settings. Well-Root™ PT’s premixed formulation simplifies application compared to light-curing cements, which require specialized equipment. Although long-term clinical data are limited, Well-Root™ PT’s success rates, comparable to those of MTA [20,21], suggest a similar clinical potential, pending further in vivo studies.

The effects of heat generation and exposure to oral fluids during ultrasonic condensation were not evaluated in this in vitro study, representing a limitation for clinical extrapolation. Ultrasonic activation can generate localized heat due to acoustic energy dissipation, potentially altering the hydration kinetics or microstructure of Well-Root™ PT, a calcium silicate-based bioceramic [49]. Excessive heat may accelerate setting but risks microcracking or increased porosity, compromising the apical seal, as observed with prolonged activation (15-s Ultrasonic group) in our study. Similarly, oral fluids such as saliva, blood, or periapical exudates could contaminate the material during placement, potentially disrupting its setting reaction or adhesion to dentin walls, which was not replicable in our standardized 3D-printed models. Prior studies suggest that calcium silicate-based materials maintain stability in moist environments, but blood contamination may increase porosity and reduce sealing efficacy [50,51]. Future studies should incorporate temperature monitoring during ultrasonication and simulate oral fluid exposure to assess their impact on Well-Root™ PT’s void formation, microhardness, and long-term sealing ability, ensuring clinical relevance for apexification procedures.

Limitations: This study utilized standardized 3D-printed plastic models to ensure consistent canal anatomy and eliminate biological variability, facilitating precise evaluation of condensation techniques. However, these models do not fully replicate the complex dentin architecture, mineral content, or thermal conductivity of natural teeth, limiting the ecological validity of the findings for clinical applications. Furthermore, the in vitro model cannot account for heat transfer during ultrasonic activation, occlusal forces under functional loading, or dentin permeability, which may influence Well-Root™ PT’s performance in vivo. Including a comparative group with extracted human teeth would have enhanced the study’s applicability to natural dentition. Direct statistical comparisons with literature data on MTA and Biodentine™ were not feasible due to variations in experimental conditions and methodologies, limiting the ability to quantitatively align Well-Root™ PT’s performance with other materials.

Incorporating multiple operators and blinding protocols would have mitigated this risk, but was not possible within the study’s constraints. A single examiner performed all micro-CT and Vickers microhardness evaluations, which may introduce analytical bias due to subjective interpretation of quantitative measures. The absence of blinding in micro-CT analysis may introduce subjective threshold bias, and the lack of temperature rise measurements during ultrasonication or simulation of clinical factors like blood and periapical tissue fluids limits insights into their effects on Well-Root™ PT’s setting and sealing properties in vivo. Furthermore, the short-term in vitro nature of this study, focusing on void formation and microhardness after one week, precludes extrapolation to long-term clinical outcomes, such as in vivo sustained sealing ability or material durability. It should be noted that measuring the microhardness at three specific points (500, 1000, and 1500 µm) along the 2 mm width of the apical plug may not fully capture its subtle variations across the entire plug.

The Vickers microhardness test provided static measurements, but did not capture dynamic loading–unloading curves, limiting quantitative analysis of elastic versus plastic deformation. Future studies using nanoindentation could provide deeper insights into the surface mechanical properties of Well-Root™ PT under varying condensation techniques. The selection of 3, 9, and 15 s for ultrasonic activation provided a broad spectrum of durations but may not fully capture the optimal activation time. Testing additional intermediate durations (e.g., 5, 7, or 12 s) could further refine the dose–response relationship and identify a precise optimal duration for clinical use.

Future research should address these limitations by using natural teeth, multiple blinded operators, in vivo assessments, and a denser or continuous sampling strategy to provide a more comprehensive assessment of material heterogeneity at different levels of the apical plug. Long-term leakage tests and comparisons with emerging calcium phosphate or hybrid cements under various condensation techniques would further elucidate Well-Root™ PT’s clinical performance relative to other bioactive materials.

## 5. Conclusions

Indirect ultrasonic activation for 3 s enhances the quality of Well-Root™ PT apical plugs by reducing void formation while maintaining high microhardness, thereby improving the apical seal in necrotic immature permanent teeth. Prolonged ultrasonic activation (15 s) should be avoided due to its detrimental effects on material integrity. These findings provide evidence-based guidance for clinicians to improve the outcomes of apical plug treatments, ultimately supporting the long-term prognosis of endodontically treated immature teeth.

## Figures and Tables

**Table 1:** Distribution of the study groups based on the condensation technique.

Groups	Number of Samples	Material and Composition	Application Technique
Group 1 Manual Condensation	12	Well-Root™ PT in a compule (0.25 g)—Calcium aluminosilicate, zirconium oxide, calcium illuminate, calcium sulfate, thickening agent.	Manual condensation—condensation of the material was performed with an endodontic plugger.
Group 2 3-s Ultrasonic	12		Indirect ultrasonic condensation by applying indirect ultrasonic activation at 25 kHz to the material for 3 s, with the shaft of the plugger in contact with the tip.
Group 3 9-s Ultrasonic	12		Indirect ultrasonic condensation by applying indirect ultrasonic activation at 25 kHz to the material for 9 s, with the shaft of the plugger in contact with the tip.
Group 4 15-s Ultrasonic	12		Indirect ultrasonic condensation by applying indirect ultrasonic activation at 25 kHz to the material for 15 s, with the shaft of the plugger in contact with the tip.

## Data Availability

The original contributions presented in this study are included in the article. Further inquiries can be directed to the corresponding author.

## Abbreviations

The following abbreviations are used in this manuscript:

MTA	Mineral trioxide aggregate
Micro-CT	Micro-computed tomography
